# Distinct effects of contrast and color on subjective rating of fearfulness

**DOI:** 10.3389/fpsyg.2015.01521

**Published:** 2015-10-08

**Authors:** Zhengang Lu, Bingbing Guo, Anne Boguslavsky, Marcus Cappiello, Weiwei Zhang, Ming Meng

**Affiliations:** ^1^Department of Psychological and Brain Sciences, Dartmouth CollegeHanover, NH, USA; ^2^Department of Psychology, University of California, RiversideRiverside, CA, USA

**Keywords:** contrast, color, emotion, signal detection theory, psychophysics

## Abstract

Natural scenes provide important affective cues for observers to avoid danger. From an adaptationist perspective, such cues affect the behavior of the observer and shape the evolution of the observer’s response. It is evolutionarily significant for individuals to extract affective information from the environment as quickly and as efficiently as possible. However, the nearly endless variations in physical appearance of natural scenes present a fundamental challenge for perceiving significant visual information. How image-level properties, such as contrast and color, influence the extraction of affective information leading to subjective emotional perception is unclear. On the one hand, studies have shown that visual perception and emotional perception seem to interact with each other at the earliest stages in cortical processing. On the other hand, it is important for high-level subjective ratings to be invariant to low-level visual properties. Using a psychophysical approach and signal detection theory (SDT), we tested how contrast and color influenced fearfulness ratings of a set of natural scene pictures that varied in contents and in levels of fearfulness. Image contrast influenced perceptual sensitivity but not the decision criterion of fearfulness rating, whereas color affected the decision criterion but not perceptual sensitivity. These results show that different low-level visual features contribute independently to sensitivity or decision criterion in affective perception, suggesting distinct interactions between visual cognition and affective processing. Specifically, our naturalistic approach using a novel stimulus set, combined with SDT, has demonstrated two dissociable types of cognitive mechanisms underlying how image-level properties leverage the extraction of affective information in natural vision.

## Introduction

Affective information in a natural scene mediates transactions in the environment that either promotes or threatens survival (Lang et al., 1997); for example, by rendering fight-or-flight responses (Whalen et al., 1998; Phan et al., 2002; Adolphs, 2013). According to the theory of biological communication, such affective cues influence the behavior of the observer and shape the evolution of the observer’s response (Scott-Phillips, 2008). It is therefore pivotal for individuals to extract affective information from the environment as fast and efficiently as possible. Many aspects of a natural scene may provide diagnostic cues to inform a dangerous situation. Contrast patterns could be highly characteristic of a poisonous spider (e.g., stripes on its back); a visually salient color (e.g., red) could be highly predictive of a violent scene (e.g., a bloody murder). The evolutionary forces (to survive) acted on the signaler (spider) to give a particular type of signal (stripes means poisonous), and also acted on the receiver to respond to that signal (to avoid being poisoned). Such “strategic design” influenced the evolution of biological communication (Guilford and Dawkins, 1991). However, it is also of interest how image-level properties of a scene would modulate our subjective evaluation of affective information extracted from diagnostic cues. For instance, would a poisonous spider with stripes on its back be evaluated as scarier in a bright-lit condition than a dim-lit condition? Dangerous stimuli (e.g., a spider or a snake) may have large variations in physical appearances under various viewing conditions. One possibility is that early perceptual processing may rely heavily on low-level visual properties to make an early judgment about the valence of a stimulus (affective prediction hypothesis; Barrett and Bar, 2009). Alternatively, affective representation may not rely on visual analyses of any particular dimension of the image-level properties as long as these properties, such as luminance, contrast, and spatial resolution, are above certain detection thresholds for serving as diagnostic cues.

Correspondingly, the literature on this issue shows mixed findings. On the one hand, visual perception and emotional perception seem to interact with each other at early stages in vision (Lebrecht et al., 2012) in that low-level visual features directly impact affective judgment. Several studies have investigated the relation between spatial frequencies and emotions using a variety of methods, stimuli, and rationales (De Cesarei and Codispoti, 2013). When comparing low-pass filtered images to intact images, low-pass filtered images were rated less arousing and less pleasing (De Cesarei and Codispoti, 2008). Moreover, when pictures were blurred at different degree (intact, intermediate blurring, maximum blurring), a reduced modulation of skin conductance was found corresponding to the degree of blurring, suggesting that emotional reactions might be modulated by image-level visual properties (De Cesarei and Codispoti, 2010). Support for the use of color in valence judgment has also been found, where positive images elicited larger P300 amplitude than negative or neutral images only for color pictures and not gray-scale pictures (Cano et al., 2009).

On the other hand, it seems important for high-level subjective ratings to remain robust to variance in low-level visual features such as position, scale, pose, and illumination (DiCarlo and Cox, 2007). Consistent with this functional necessity of surface-feature invariance for emotional perception, some research demonstrated that image-level properties of affective pictures, such as picture size, spatial frequency, brightness and complexity, did not change affective responses measured by event-related potentials (ERPs) (Junghofer et al., 2001; De Cesarei and Codispoti, 2006, 2011a,b). In addition, removing color information shows no influence on the valence modulation of the late positive potentials (LPPs), which are larger for pleasant and unpleasant pictures than neutral pictures, implying that the processing of emotional contents does not critically rely on color information (Codispoti et al., 2012; see also Junghofer et al., 2001; Weymar et al., 2009 for different paradigms with similar results). The conflicting results of these studies might be partially driven by various measurements of emotional responses used in the experiments. However, different components of emotional responses, as reflected by different measurements, might also be modulated by different low-level visual properties. For example, spatial frequency could modulate both subjective ratings and skin conductance (De Cesarei and Codispoti, 2008, 2010); but picture size, spatial frequency and color did not modulate ERP responses (De Cesarei and Codispoti, 2006, 2011a,b; Codispoti et al., 2012).

We argue that the conflicting results of previous studies could be reconciled by examining how image-level properties of stimuli would affect different aspects of the perception of emotional information. First, to simplify the investigation, we focused on the perception of fearful stimuli, because fear is a basic emotion and crucial survival mechanism that has been studied extensively (e.g., Bradley et al., 2001; Cardinal et al., 2002), although we believe that the same approach could be used to test other dimensions of affective perception. Second, to examine different aspects of perceiving fearful stimuli, signal detection theory (SDT) will be applied to the data analysis. It has been well established that SDT can be used to estimate the two different aspects of perception: sensitivity and decision criterion (Green and Swets, 1966). According to SDT, perceptual decisions are based on the strength of a perceptual signal in relation to a decision criterion, which is a continuous process of information accumulation (Wixted, 2007). Methodological approaches that apply SDT have therefore been used to examine subjective experiences of perception, such as pain assessment (Naliboff et al., 1981; Classen, 1984; Kirwilliam and Derbyshire, 2008), distinctions between one’s own correct and incorrect decisions (Galvin et al., 2003), subjective confidence ratings of the correctness of a discrimination response (Zehetleitner and Rausch, 2013), subjective confidence in reporting the absence of a stimulus (Kanai et al., 2010), subjective assessment of the quality of the percept (He et al., 2009), and subjective awareness of fearful faces (Szczepanowski and Pessoa, 2007), in addition to the events defined independently of the observer. To the best of our knowledge, however, no one has previously taken a SDT approach to investigate how low-level visual properties may modulate affective ratings.

In order to apply SDT to tease apart different aspects of subjective rating of fearfulness (i.e., true perceptual effects in fearful rating resulting from visual properties), images with a wide range of levels of fearfulness are needed. International Affective Picture System (IAPS) is a very useful standardized collection of color photographs of objects and scenes across a wide range of categories (Lang et al., 2008). Typically, studies that tried to investigate relationships between visual processing and emotion often selected pleasant, neutral or unpleasant subsamples from this image database (e.g., Bradley et al., 2007). However, subsampling the fearful stimuli from this database contains only extreme fearful images, which are rarely seen in a naturalistic environment, but contains no images with intermediate levels of fearfulness between unpleasant and neutral, which only allows measurements of one extreme level of fearful ratings that cannot be used for SDT analyses. In the present study, we compiled a new stimulus set that contains images resembling what everyone may see from a daily routine of life. Importantly, this set of stimuli contains not only the most fearful stimuli but also pictures widely varied in different fearful levels, which therefore provide the opportunity for applying SDT to disentangle how image-level properties may differently influence affective perception of scene pictures in a fine-grained manner.

Specifically, by taking the SDT approach, we examined effects of contrast and color on the subjective rating of fearfulness. It is known that information in different spatial frequency bands plays significantly different roles in recognizing fearful facial expressions (Vuilleumier et al., 2003; Pourtois et al., 2005; Mermillod et al., 2009, 2010a). Given that contrast sensitivity is intrinsically modulated as a function of spatial frequencies (Wandell, 1995), we can expect that visual contrast may affect the detection of emotion information (cf. Pallett and Meng, 2013). Nonetheless, how contrast may affect the detection of emotion information in scene pictures has not been tested. Similarly, color plays a critical role at multiple levels of visual processing (Gegenfurtner and Rieger, 2000; Tanaka et al., 2001). Color can facilitate object recognition regardless of the diagnostic value of color for the identification of the object (Wurm et al., 2004). However, how color information might affect subjective evaluations of fearfulness in natural scene pictures is still unclear.

## Materials and Methods

### Participants

Participants were 109 students from Dartmouth College. Among them, 56 students (mean age = 19 years, 43 females, 29 Caucasians) participated in experiment 1 to test the effect of contrast. The other 53 students (mean age = 19 years, 34 females, 32 Caucasians) participated in experiment 2 to test the effect of color. The sample size was comparable with that of similar previous studies (e.g., De Cesarei and Codispoti, 2008; Nummenmaa et al., 2010; Pilarczyk and Kuniecki, 2014). The participants for both experiments were representative in the same manner of the psychology major undergraduate population at Dartmouth College with respects to age, gender and ethnicity. All participants had reported normal color vision and normal or corrected to normal visual acuity. They were not informed the purpose of the experiments in advance. They had not participated in any experiments using the same set of stimuli. Participants received course credit. All gave informed consent. This research was approved by the Committee for the Protection of Human Subjects at Dartmouth College and conducted in accordance with the 1964 Declaration of Helsinki.

### Materials

One Hundred and fifty-seven colorful pictures devoid of human faces were collected from the Internet, which displayed a wide range of content and fearful levels, from animals, human body parts, objects (knives, weapons), acrophobia scenes, landscapes of cities and streets, dark lit natural scenes to burning buildings, cars and forests. First, all images with original color were converted to gray scale by eliminating the hue and saturation information while retaining the luminance information. The color and gray scale images were used as stimuli in the color experiment. Next, based on the gray scale images, two more versions were generated with either a higher level of contrast (mean luminance = 126, on the scale from 0 = black to 255 = white; Root Mean Square (RMS) contrast = 84) or a lower level of contrast (mean luminance = 126, RMS contrast = 10) than the original gray scale images, and were used in the contrast experiment. The image processing was performed using MATLAB and SHINE toolbox (Willenbockel et al., 2010). The size of stimuli is 400 × 400 pixels for all of the color, gray scale, high contrast, and low contrast images.

In order to obtain a consensual judgment for each of the 157 images, an independent group of 43 participants (mean age = 19 years, 32 females, 23 Caucasians) were tested. This group of participants was recruited in the same manner as the main experiments, representative of the psychology major undergraduate population at Dartmouth College with respects to age, gender and ethnicity. A large version (600 × 600 pixels) and a small version (200 × 200 pixels) of the stimuli in gray scale were rated for fearfulness on a 7-point scale (1 = not scary at all, 7 = extremely scary) with similar experimental procedures as the main experiments (detailed below). The ratings for each of the 157 images were averaged across large and small stimuli and then across participants as the consensual judgment for each image. Please see supplementary materials for the distribution of averaged ratings of the 157 images.

### Procedure

A between-subject and within-subject mixed design was used to examine the effect of contrast or color on the judgment of fearfulness. In the contrast experiment, participants were randomly divided into two groups: (1) viewing high contrast images first and then low contrast images; (2) viewing low contrast images first and then high contrast images. Two blocks of stimuli were presented for each group with the same low-level feature (e.g., high-contrast or low-contrast) within each block. In the color experiment, stimuli were presented in a color images block and a gray images block. In all other respects, the two experiments were designed in the same manner.

Using MATLAB with Psychtoolbox (Brainard, 1997; Pelli, 1997; Kleiner et al., 2007), images were presented on a gray background at the center of a 21-inch Dell P1130 CRT monitor with a refresh rate of 85 Hz at a viewing distance of about 65 cm. Each image had 32bit color depth and spatial resolution of 1280 × 1024 pixels. For a typical block, participants were first familiarized with the stimuli by passively viewing all of the stimuli for 1 s each without any inter-stimulus interval. A red fixation cross was always presented in the center of the screen. They were then shown the images one at a time, and were asked to rate the fearfulness of the image on a scale from 1 to 7 (1 = not scary at all, 7 = extremely scary). Each image was presented once in each block and remained on screen until a rating was made. There was a 700 ms inter-trial interval. The order of presentation of the images was random and was different for each participant.

### Data Analysis

We conducted conventional ANOVA for all the rating data collected in experiments 1 and 2, except that 3 trials from 1 subject in experiment 2 were excluded because of mistaken keyboard presses. Based on the consensual judgment (averaged rating) in the independent rating experiment, the 157 images were divided into 5 fearful levels with nearly matching number for each level: Level 1 included 32 images (range of mean ratings [1.38, 2.19]); level 2 included 31 images ([2.19, 2.74]); level 3 included 32 images ([2.74, 3.38]); level 4 included 32 images ([3.38, 4.08]); level 5 included 30 images ([4.08, 5.34]). In total, there were 20 conditions (5 fearful levels × 2 levels of image feature × 2 presentation orders) in each data set. Three-way (5 × 2 × 2) mixed design ANOVA was then used for analyzing the effects of fearfulness level (5 levels: 1–5) and contrast or color (two levels: low/high contrast or color/gray) as within-subject factors, and presentation order [two levels: first present low contrast (or color) and then high contrast (or gray) vs. first present high contrast (or gray) and then low contrast (or color)] as a between-subject factor. Effect size indicator, η^2^ (generalized eta squared) was computed to further evaluate these effects on the subjective fearful ratings.

For the SDT based analysis, a median (3.05) was first computed using the consensual judgment (averaged rating) data of all the stimuli obtained in the independent rating experiment. Second, consensual judgment data were split using the median to fearful and non-fearful stimuli. For every fearful stimulus (signal), each rating in the color and contrast experiment was classified as either a hit, if it was higher than its consensual judgment, or a miss, if it was lower than its consensual judgment; similarly, for every non-fearful stimulus (noise), each rating in the color and contrast experiment was classified as either a false alarm, if it was higher than its consensual judgment, or a correct rejection, if it was lower than its consensual judgment. Using these hit and false alarm rates, an overall decision criterion (c) were computed for each condition and each observer.

Receiver operating characteristics (ROCs) were then constructed for the contrast and color experiment separately, by plotting p(hit) and p(FA) across variations in response criterion or confidence on the *y*-axis and *x*-axis, respectively. The p(hit) is the proportion of rating responses greater than a given criterion for fearful stimuli (signal); and the p(FA) is the proportion of rating responses greater than the criterion for non-fearful stimuli (noise). For example, the first (leftmost) data point represents the proportion of rating responses of 7 for fearful and non-fearful stimuli on the *y*-axis and the *x*-axis, respectively, which were accustomed hit and false alarm rates using the most conservative decision criterion (i.e., only ratings of 7 for fearful stimuli were counted as hits). The next data point on ROCs represents accustomed accumulative hit and false alarm rates for pictures eliciting fearfulness rating of 7 and 6, using a less conservative decision criterion (i.e., ratings of 6 and higher were counted as hits). Keep relaxing decisional criteria will eventually lead to 100% of accumulative hit and false alarm rates for pictures eliciting fearfulness rating from 1 to 7, which by convention is not plotted. Overall six data points on ROCs were plotted to represent the accumulative p(hit) and p(FA) for the 7-point rating scale by adjusting decisional criterion. The ROCs were then fit with equal-variance Signal Detection theory (EVSD) (Macmillan and Creelman, 1991) using a maximum likelihood estimation procedure (Myung, 2003). This procedure yielded one parameter, discriminability measured as d’, for each condition and each observer. Overall, the EVSD model provides a good fit for the data, accounting for more than 95% of variance on average.

## Results

**FIGURE 1:**
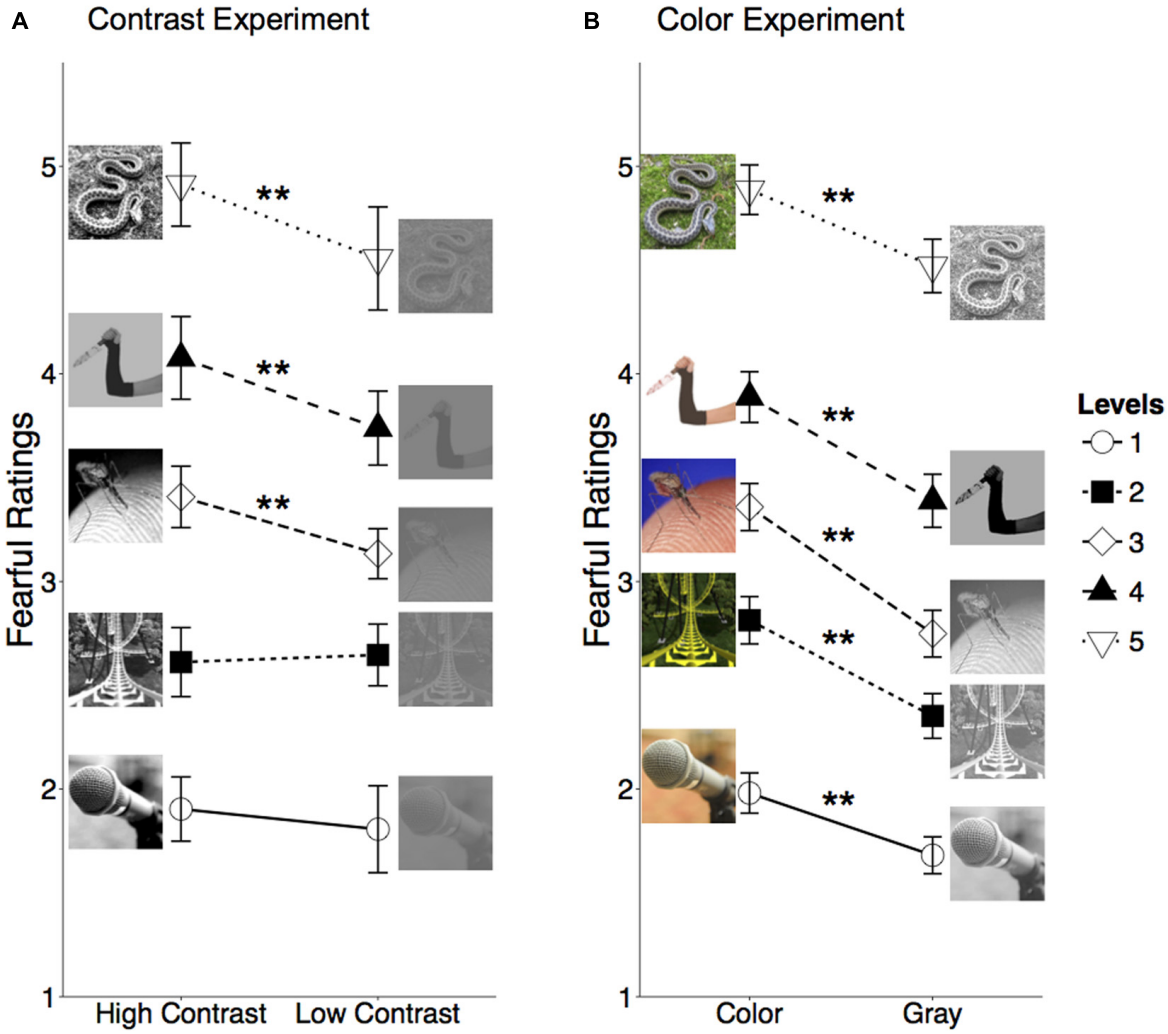
**Mean fearful ratings for (A) high contrast and low contrast images at five levels of fearfulness in the contrast experiment and (B) color and gray images at five levels of fearfulness in the color experiment.** Error bars represent 95% confidence intervals. Examples of stimuli at each level of fearfulness are shown next to the corresponding data point for each experimental condition. ^∗∗^indicates *p* < 0.01, Bonferroni corrected.

In the contrast experiment, significant main effects of fearful level and contrast were found [*F*(4,216) = 194.61, *p* < 0.001, η^2^ = 0.44; *F*(1,54) = 12.01, *p* < 0.01, η^2^ = 0.01, respectively]. The interaction between presentation order (rating block) and contrast [*F*(1,54) = 7.01, *p* = 0.01, η^2^ = 0.01], as well as the interaction between fearful level and contrast [*F*(4,216) = 8.00, *p* < 0.001, η^2^ = 0.003] were also significant. Moreover, a significant three-way interaction was found [*F*(4,216) = 4.98, *p* < 0.01, η^2^ = 0.002], which suggests that the presentation order may modulate the effect of contrast differently at various levels of fearfulness. In the color experiment, the main effects of fearful level and color/gray were found to be significant [*F*(4,204) = 204.06, *p* < 0.001, η^2^ = 0.43; *F*(1,51) = 8.77, *p* < 0.01, η^2^ = 0.006, respectively]. None of the two-way interactions was significant (all *F*s < 2.34, all *p*s > 0.05). The three-way interaction was not significant either [*F*(4,204) = 1.63, *p* = 0.17, η^2^ = 0.0001]. According to our signal detection hypothesis, differentiating fearful levels of the stimuli would be different for the second time viewing of the stimuli, because memories of the previously viewing of the stimuli may be informative (i.e., providing additional signals) for observers to rate the stimuli. While the discrepancy of three-way interactions between the two experiments is consistent with possible different perceptual effects of color and contrast, it might also be caused by different memory effects or a combination of perception and memory, which exceeds the scope of the present paper. To avoid any possible confounding effects due to non-perceptual factors, we focus on between-subject comparisons for data collected in the first block of each experiment (**Figure 1**).

As shown by **Figure 1A**, in the contrast experiment, a two-way ANOVA for the first rating block reveals that the main effect of fearful level was significant [*F*(4,216) = 194.93, *p* < 0.001, η^2^ = 0.47]. However, the main effect of contrast was not significant [*F*(1,54) = 1.22, *p* = 0.27, η^2^ = 0.02]. The interaction between contrast and fearful level was not significant either [*F*(4,216) = 1.99, *p* = 0.10, η^2^ = 0.009]. Pair-wise comparisons were then conducted to examine the contrast effect at different fearful levels. Significant effects of contrast on fearful ratings were found at levels 3, 4, and 5 (all *p*s < 0.01, Bonferroni corrected), whereas the effect of contrast was not significant at levels 1 and 2 (*p*s > 0.4, Bonferroni corrected). As shown by **Figure 1B**, in the color experiment, a two-way ANOVA for the first rating block reveals that the main effect of fearful level was significant [*F*(4,204) = 191.57, *p* < 0.001, η^2^ = 0.45]. The main effect of color was also significant [*F*(1,51) = 5.26, *p* = 0.03, η^2^ = 0.07]. The interaction between color and fearful level was not significant [*F*(4,204) = 1.07, *p* = 0.37, η^2^ = 0.005]. Pair-wise comparisons were then conducted to examine the color effect at different fearful levels. The effects of color on fearful ratings were found to be significant at all five fearful levels (all *p*s < 0.01, Bonferroni corrected).

**FIGURE 2:**
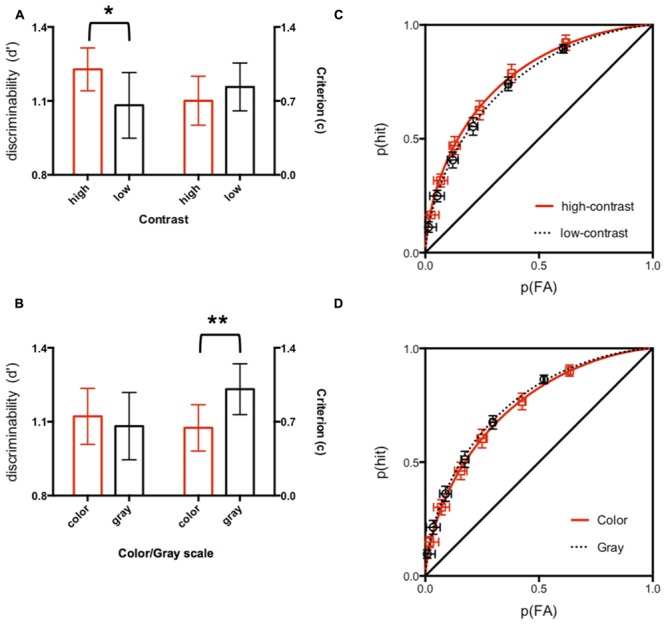
**(A,B)** The discriminability (plotted on left *y*-axis) and criterion (plotted on right y-axis) extracted from ROCs **(C,D)** from aggregated data for the two experiments. ^∗^indicates *p* < 0.05, ^∗∗^indicates *p* < 0.01.

**Figure 2** shows the results of the SDT analyses. The ROC curve for the low contrast condition fell below that of the high contrast condition (**Figure 2C**), suggesting that overall discrimination of fearfulness was greater for the high contrast stimulus. This was confirmed as significantly higher d’ for the high contrast stimuli compared to low contrast stimuli (*t*(54) = 1.874, *p* = 0.03, 95% confidence interval for the difference between means [0.01, 0.30], **Figure 2A**). No significant difference was found in overall decision criterion, c (*t*(54) = 0.75, 95% confidence interval for the difference between means [-0.43, 0.20]). In sharp contrast, each data point on the ROCs showed a rightward shift for the color condition compared to corresponding data point for the gray condition (**Figure 2D**). This was confirmed as significantly higher overall decision criterion, c, for the color stimuli compared to gray stimuli (*t* (51) = 2.31, *p* = 0.01, 95% confidence interval for the difference between means [-0.68, -0.05], **Figure 2B**). No significant difference was found in d’ (*t* (51) = 0.22, 95% confidence interval for the difference between means [-0.15, 0.19]).

## Discussion

The nearly endless variations of visual properties in natural scenes present a fundamental challenge for abstracting and perceiving coherent relevant information in the environment. To maintain representational constancy, object and scene perception should be robust to variations in image-level properties (DiCarlo and Cox, 2007; Gilchrist, 2012). However, some of these variances in natural stimuli are pivotal for detecting emotional information. Our results indicate that both image contrast and color modulate subjective fearfulness ratings of scene pictures. This is consistent with previous studies suggesting that affective perception is not isolated from early visual processing of color, size, resolution, brightness, spatial frequency of the stimuli pictures (Vuilleumier et al., 2003; Pourtois et al., 2005; De Cesarei and Codispoti, 2006, 2008, 2010; Mermillod et al., 2010b; Codispoti et al., 2012; Lakens et al., 2013). Yet why would image-level visual properties influence ratings of fearful scenes? The present results suggest that different mechanisms could support the effects of visual features on fear perception.

As suggested by our SDT analyses, contrast influences perceptual sensitivity but not decision criterion of rating fearfulness in the scene pictures. By contrast, color shifts the decision criterion without affecting perceptual sensitivity for rating fearfulness in natural scene pictures, suggesting that color information is less effective in modulating the sensitivity of fearful perception than stimulus contrast. However, this does not necessarily mean that color is less effective in influencing subjective rating. In fact, the criterion effect of color increased overall fearfulness rating. Note that we would have not found these differences, had we only conducted conventional ANOVAs. Results of ANOVAs suggest that both contrast and color information affected fearful ratings. When rated for the first time, the effect of contrast was stronger for the images of medium to high levels of fearfulness (levels 3–5 in our study) than for the neutral (level 1) or nearly neutral images (level 2). When rated for the first time, the effect of color was highly significant at all levels of fearfulness. These results are consistent with what our SDT analyses revealed. However, without applying the SDT, it would have been difficult to understand how these different effects were caused by sensitivity or detection criterion differences.

Our findings also reconcile previously reported mixed results regarding the role of color on affective perception (Cano et al., 2009; Codispoti et al., 2012). On the one hand, color does not improve the sensitivity of discriminating fearful and non-fearful stimuli, thus leading to little effect on the neural marker of valence modulated LPP (Codispoti et al., 2012). On the other hand, changes in decision criterion due to color can still be accompanied by changes in P300, which is an ERPs component elicited in the process of decision making (Cano et al., 2009). Note that the effect of color on decision criterion observed in the present study does not exclude possible modulations of perceptual sensitivity in discriminating emotional stimuli under extreme cases. For example, the presence of a red splash of blood in an image could lead to rapid detection of danger. When color is diagnostic of a scene category, it mediates express scene recognition (Oliva and Schyns, 2000), e.g., red may signify blood. Therefore, in this case, color plays a diagnostic role in the operation of high-level vision: categorizing a scene into fearful or not fearful (Tanaka et al., 2001). However, such effect of color for particular diagnostic contents of images may be negligible in the present study because a wide range of contents with large variance in the level of fearfulness, from animals, human body parts, weapons/objects to various scenes, including street and city which are not color-diagnostic (Oliva and Schyns, 2000), were used. Therefore, the present results are more representative for interactions between emotional perception and visual perception in natural vision, regardless of the specific diagnostic value of color for scene recognition in any given stimulus.

The dissociable effects of image color and contrast on fearfulness rating may arise from interactions between perception and metaphor. A recent study demonstrated that stimulus brightness could influence affective rating in that brighter images tended to be rated more positive whereas dimmer images tended to be rated more negative (Lakens et al., 2013). Similarly, interactions between metaphoric understanding and embodied cognition (Gibbs, 1994; Gibbs et al., 2004; Xie and Zhang, 2014) can lead to a metaphoric association between stimulus contrast and affective perception. That is, high-contrast and low-contrast can metaphorically correspond to being more fearful and less fearful, respectively. By contrast, given the lack of any metaphoric association between stimulus color and affective judgment, the effects of color on affective judgment are largely driven by the change of decision criterion.

Besides extending our understanding of the influences of visual properties on affective perception, the current study has provided some methodological tools for research on affective perception. The fearful stimulus set developed in the present study has a wide range of fearfulness levels and can be controlled on certain low-level properties. With this set, SDT methods can be used to tease apart sensitivity and decision criterion, two independent factors contributing to fear perception. The dissociable effects on these two factors would have been impossible to see if we had only compared images that are either very fearful or not fearful at all. SDT methods may be used to resolve the variety of previous findings on whether certain visual properties, such as spatial frequency information, influence affective processing (Vuilleumier et al., 2003; De Cesarei and Codispoti, 2008). Future studies using SDT approaches can extend current findings by applying type 2 task and parametric manipulations of image-level properties to further investigate how metacognitive (type 2) sensitivity (e.g., Maniscalco and Lau, 2012) to affective information would be modulated by graded levels of image-level properties. Moreover, it is important to note that subjective rating used in the present study is prone to biases from beliefs and expectation. Therefore, it would be interesting for future studies to generalize the present findings with more objective measures of emotional responses, such as skin conductance response and neural activities in amygdala using functional imaging. Nevertheless, by showing that low-level properties such as contrast and color affect different aspects of the subjective rating of fearful scenes, our findings provide a premise for future studies to investigate how danger may be detected from natural scenes based on cognitive algorithms computing the visual properties of stimuli.

## Conflict of Interest Statement

The authors declare that the research was conducted in the absence of any commercial or financial relationships that could be construed as a potential conflict of interest.
