# Supplementary material for: Distinct effects of contrast and color on subjective rating of fearfulness
Source: Front Psychol. 2015 Oct 8;6:1521. doi: 10.3389/fpsyg.2015.01521 (PMC4597033; doi:10.3389/fpsyg.2015.01521)
Supplement: Supplementary file 1 [file Image_1.PDF]

Averaged rating distribution in the independent rating experiment

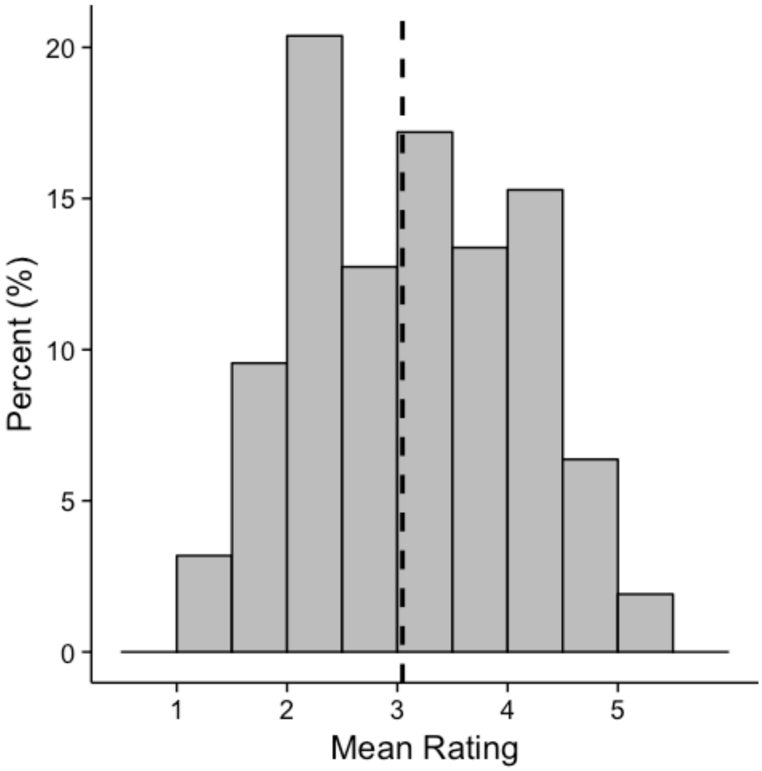

Distribution of averaged ratings for all of 157 stimuli in the independent rating experiment. The vertical dash line represents the median (3.05).
